# Slow darkening of pinto bean seed coat is associated with significant metabolite and transcript differences related to proanthocyanidin biosynthesis

**DOI:** 10.1186/s12864-018-4550-z

**Published:** 2018-04-16

**Authors:** Kishor Duwadi, Ryan S. Austin, Hemanta R. Mainali, Kirstin Bett, Frédéric Marsolais, Sangeeta Dhaubhadel

**Affiliations:** 1 London Research and Development Centre, Agriculture and Agri-Food Canada, 1391 Sandford Street, London, ON N5V 4T3 Canada; 2 Department of Biology, University of Western Ontario, London, ON Canada; 3 Department of Plant Sciences, University of Saskatchewan, Saskatoon, SK Canada

**Keywords:** Pinto bean, Postharvest seed coat darkening, Proanthocyanidins, Transcriptome, Phenylpropanoids

## Abstract

**Background:**

Postharvest seed coat darkening in pinto bean is an undesirable trait resulting in a loss in the economic value of the crop. The extent of darkening varies between the bean cultivars and their storage conditions.

**Results:**

Metabolite analysis revealed that the majority of flavonoids including proanthocyanidin monomer catechin accumulated at higher level in a regular darkening (RD) pinto line CDC Pintium than in a slow darkening (SD) line 1533–15. A transcriptome analysis was conducted to compare gene expression between CDC Pintium and 1533–15 and identify the gene (s) that may play a role in slow darkening processes in 1533–15 pinto.

RNAseq against total RNA from RD and SD cultivars found several phenylpropanoid genes, metabolite transporter genes and genes involved in gene regulation or modification to be differentially expressed between CDC Pintium and 1533–15.

**Conclusion:**

RNAseq analysis and metabolite data of seed coat tissue from CDC Pintium and 1533–15 revealed that the whole proanthocyanidin biosynthetic pathway was downregulated in 1533–15. Additionally, genes that encode for putative transporter proteins were also downregulated in 1533–15 suggesting both synthesis and accumulation of proanthocyanidin is reduced in SD pintos.

**Electronic supplementary material:**

The online version of this article (10.1186/s12864-018-4550-z) contains supplementary material, which is available to authorized users.

## Background

Postharvest darkening of seed coat is a concern for dry bean (*Phaseolus vulgaris* L.) producers worldwide. The seed coat of many beans such as pinto, cranberry and red beans darken during aging which affects their visual quality, leading to a decrease in consumer preference due to which the common bean producers, exporters and vendors encounter significant loss in crop value. Studies have indicated that the postharvest darkening phenomenon is attributed to a combination of environmental factors such as elevated temperatures, humidity, exposure to light [1, 2] as well as crop genetics [3, 4]. There are at least 3 phenotypes of bean seed coats that respond differently to aging as identified by various common bean breeding programs: regular darkening (RD), slow darkening (SD) and non-darkening (ND) [3].

**Fig. 1:**
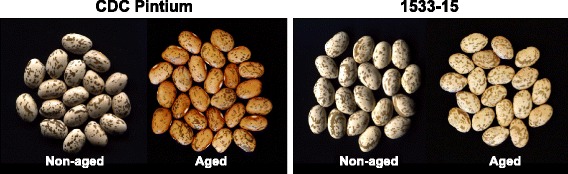
Photographs illustrating postharvest seed coat darkening in pinto bean. Pictures were taken for pinto bean lines CDC Pintium and 1533–15 immediately after harvest (non-aged) and six months after storage at room temperature (aged)

**Fig. 2:**
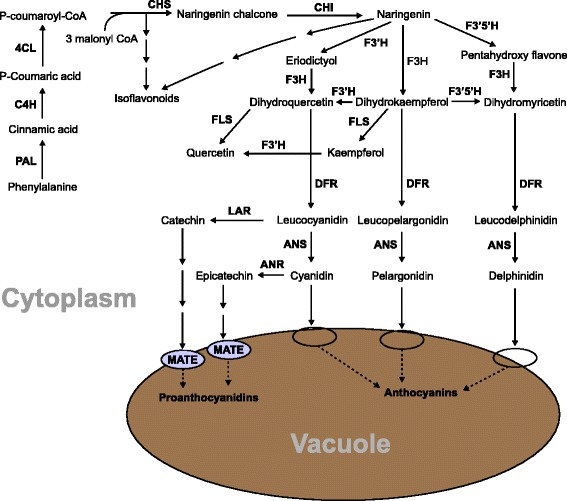
Proposed proanthocyanidin biosynthetic pathway in pinto bean seed coats. The multiple arrows indicate multiple steps and dashed arrows indicate speculative steps. The ovals indicate membrane transporters. PAL, phenylalanine ammonia lyase; C4H, cinnamate-4-hydrolase; 4CL, 4-coumarate-CoA ligase; CHS, chalcone synthase; CHR, chalcone reductase; CHI, chalcone isomerase; F3H, flavanone 3-hydroxylase; F3’H, flavonoid 3′-hydroxylase; F3’5’H, flavonoid 3′,5′-hydroxylase; DFR, dihydroflavonol reductase; ANS, anthocyanidin synthase; FLS, flavonol synthase; LAR, leucoanthocyanidin reductase; ANR, anthocyanidin reductase; ANS, anthocyanidin synthase; MATE, multidrug and toxic compound extrusion protein

Among the market classes of dry beans, postharvest seed coat darkening has been a major issue in pintos. CDC Pintium (RD) and 1533–15 (SD) are two pinto bean cultivars released by the Crop Development Centre at the University of Saskatchewan. These two pinto lines have been used in several studies to investigate the phenomenon of seed coat darkening. The seed coat of CDC Pintium turns from creamy white to brown within six months of normal storage whereas the seed coat of 1533–15 turns from creamy white to a stable light brown over the same duration of time (Fig. 1). Arabidopsis mutants with changes in seed coat color and cranberry beans with susceptibility to postharvest darkening show alteration in proanthocyanidin content [5, 6]. Proanthocyanidins are oligomeric flavonoids composed primarily of catechin and epicatechin units (Fig. 2). Synthesis of proanthocyanidin shares the flavonoid pathway with anthocyanins until leucocyanidin/cyanidin. Genes involved in the conserved flavonoid pathway have been well characterized in Arabidopsis using *transparent testa* (*tt*) mutants [7, 8]. A study comparing seed coat flavonoids in aged and non-aged seeds of CDC-Pintium and 1533–15 found significantly higher levels of proanthocyanidins in CDC-Pintium compared to 1533–15, where kaempferol was the main flavonol monomer in the aged and non-aged seed coats of both lines [9]. A kaempferol-catechin adduct was also identified, whose content increased during aging in CDC Pintium, suggesting that it is formed during the process of oxidation. These results were extended by a segregation analysis for RD and SD traits in recombinant inbred lines (RIL) developed from a cross between CDC Pintium and 1533–15 [10]. The SD phenotype was found to be significantly associated with reduced levels of kaempferol and polyphenol oxidase activity, the latter of which is responsible for the oxidation of polyphenols.

An attempt to study the genetics of postharvest seed coat darkening has suggested that this trait is controlled by at least two unlinked genes in dry beans. The *J* locus determines the tendency to darken such that a homozygous recessive *(jj)* results in an ND phenotype. The gene *J* is epistatic to a second major gene *Sd,* that determines how rapidly a seed coat will darken [3]. A simple sequence repeat (SSR) assay in a RIL mapping population generated from CDC Pintium and 1533–15 placed the *Sd* gene between the SSR markers Pvsd-1157 and Pvsd-1158 on chromosome 7 [11]. However, the possibility of this trait being regulated by a number of other genes cannot be overlooked. These genes could either be present on the same chromosome as the SSR markers or on different chromosomes. A detailed study of the seed coat transcriptome across seed developmental stages could shed light on the genetic factors affecting seed coat darkening.

Here we apply a transcriptomic analysis to identify genes that are expressed differentially in CDC Pintium and 1533–15 during seed coat development and investigate their potential role in the active accumulation of proanthocyanidins and/or their regulators. Genes that might have roles in the translocation of proanthocyanidin monomers/oligomers from cytoplasm to vacuoles or vacuoles to apoplast have also been identified, which potentially provides further insight into seed coat darkening and assists in the selection of candidate genes. Our study identifies several phenylpropanoid and transporter genes that are differentially expressed (DE) between RD and SD cultivars and may be key genes in determining postharvest seed coat darkening in pinto beans.

## Methods

### Plant materials

Pinto bean (*Phaseolus vulgaris* L.) cultivars CDC Pintium and 1533–15 were used for the study. CDC Pintium is a rapid darkening, early maturing pinto bean cultivar developed by the University of Saskatchewan. It has a good seed coat color at harvest which turns from creamy white to dark brown in storage. 1533–15 is a slow darkening, early maturing F_4−_derived line developed from a cross between CDC Pintium and a breeding line SC11743–3 from the International Centre for Tropical Agriculture, and registered as CDC WM-1 [9]. Its seed coat turns from creamy white to light brown at a much slower rate than CDC Pintium. Both cultivars take 20-21 days for flowering.

To collect seed coat samples, CDC Pintium and 1533–15 were grown in Pro-Mix PGX in a growth chamber under a 16 h light at 25 °C and 8 h dark at 20 °C cycle with 70–80% relative humidity. Light intensity was maintained at 300–400 μmol photons/m^2^/s. Developing seeds were harvested at four different developmental stages (30, 50, 150, 350 mg seed weight) at the same time of the day (2 pm), and their seed coats collected. The seed coat tissues were immediately frozen in liquid nitrogen and stored at − 80 °C.

### RNA extraction

Total RNA was extracted from pinto bean seed coat tissues (30, 50, 150, 350 mg seed weight) using a modified LiCl method [12]. RNA samples were quantified using a NanoDrop ND-1000 spectrophotometer (Thermo Scientific, USA), and their integrity checked using a 2100 Bioanalyzer (Agilent Technologies, USA).

### RNAseq and data analysis

The seed coat mRNA from CDC Pintium and 1533–15 (150 mg stage) was sequenced using a HiSeq2000 (Illumina Inc., USA) at the National Research Council (Saskatoon, Canada). Four biological replicates per cultivar were sequenced using 100 bp paired-end runs. Additional adapter trimming (additional to that performed by the sequencer) was performed using a custom Perl script against adapter sequences identified using FastQC. Reads from each biological replicate were mapped to representative transcripts of the *P. vulgaris* genome (Phytozome release V2.1, https://phytozome.jgi.doe.gov/pz/portal.html#!info?alias=Org_Pvulgaris, “PrimaryTranscripOnly” file) [13] using BWA with 3′ end trimming (base quality > 30) [14]. SAMtools was then used to filter PCR duplicates, remove reads with low mapping quality (Q < 20) and extract the total number of uniquely mapped reads per transcript [15]. Read counts from SAMtools were imported into R and normalized using the DESeq “counts” function. Low expressing genes possessing one or zero read counts when summed across all treatments were filtered by removing the bottom 10% quantile of genes using the R quantile function. These low expressors introduce division by zero errors in downstream analysis and are therefore dropped. Differential expression was assessed using the negative binomial test (FDR < 0.001) of DESeq [16]. Heatmaps were generated in R using the heatmap.2 function.

### Gene ontology enrichment and analysis

DE genes were subjected to Gene Ontology (GO) enrichment analysis using the Singular Enrichment Analysis tool available on AgriGO v2.0 (http://bioinfo.cau.edu.cn/agriGO/analysis.php) with a significance level of 5% using Fisher statistical testing and Yekutieli multi-test adjustment.

### Quantitative RT-PCR

Total RNA from each sample was treated with DNaseI to remove contaminating DNA using the TURBO DNA-free™ kit (Life Technologies, USA). Total RNA (1 μg) from each sample was used for cDNA synthesis using the Thermoscript™ RT-PCR System (Invitrogen, USA). For quantitative RT-PCR, SsoFastTM EvaGreen® Supermix (Bio-Rad, USA) was used with the CFX96 real-time PCR detection system (Bio-Rad, USA). The primer combination and amplicon sizes are shown in Additional file 1: Table S1. The amplicon was cloned into pGEM-T Easy vector (Promega, USA), and its sequence verified. *P. vulgaris ubiquitin* (*Phvul.007G052600*) was used as a reference gene for data normalization and to calculate the relative mRNA levels. Two biological replicates per cultivar and three technical replicates per biological replicates were used for qPCR analysis using CFX Manager (Bio-Rad, USA).

### Extraction and analysis of flavonoids

Extraction of polyphenols from pinto bean seed coat was carried out according to Hu et al. [17] with some modifications at The Metabolomics Innovation Centre, University of Alberta. The ground seed coat powder (1 g) samples were extracted in 10 mL of methanol: water (80:20 *v*/v) containing 1% HCl. The samples were first sonicated at ambient temperature for 30 min, then at 40 °C for another 30 min followed by incubation in boiling water bath for 30 min with regular vortexing. The extracts were cooled, shaken at 300 rpm for 4 h and centrifuged at 3000 rpm for 20 min at ambient temperature. The supernatant was filtered under vacuum at room temperature and the filtrate lyophilized after purging under nitrogen gas for 30 min. The lyophilized powder was dissolved in 25% methanol.

HPLC analysis of the methanol extracts was performed using an Agilent G1311 A quarternary 1100 series HPLC pump (Agilent Technologies), a Synergi RP-polar (250 × 4.6 mm, 4 μm) C18 column (Phenomenex) connected to an Agilent G1315B diode array detector. Data were collected and analyzed using ChemStation software (Agilent Technologies). Phenolic compounds in the extracts were analyzed using the reference HPLC method [18] with gradient elution program [solution A, 50 mM sodium phosphate pH 2.5 (by the addition of 85% ortho phosphoric acid), solution B, 100% methanol]: 0 min, 5% B; 15 min, 30% B; 40 min, 40% B; 60 min, 50% B; 65 min, 55% B; 80 min, 100% B; 85 min, 5% B and 90 min, 5% B. The flow rate was 1.0 mL/min and injection volume was 40 μL. Absorbance was detected at 254, 280, 306 and 340 nm using the Agilent diode array detector. Phenolic compounds in the extracts were identified by comparison of their retention times with the spectra of known polyphenol standards and quantified by using the calibration curve for each phenolic compound obtained by peak areas from the chromatogram.

## Results and discussion

### Flavonoid analysis of immature seed coat of CDC Pintium and 1533–15

**Fig. 3:**
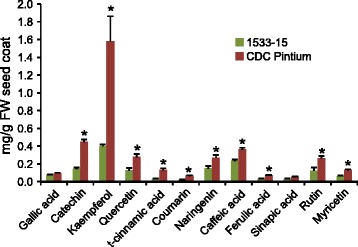
Flavonoid analysis in seed coat of pinto bean lines. Seed coat tissue was collected from 150 mg stage of developing seeds from CDC Pintium and 1533–15, and flavonoids were determined by HPLC. Data shown are mean values from three independent experiments. Asterisks (*) indicate significant difference between the samples as determined by Student’s *t*-test

A previous analysis of seed coat polyphenol levels from aged and non-aged mature seeds showed increased levels of proanthocyanidins in CDC Pintium compared to 1533–15, indicating that the difference in polyphenol levels may be linked to the postharvest seed coat darkening in CDC Pintium [9]. Since active accumulation of proanthocyanidins occurs in common bean [19] and *Arabidopsis thaliana* immature seeds [20], and intermediate/mature seed coat in cranberry beans [6], we used seed coat collected from the intermediate (150 mg) stage of developing seeds for flavonoid analysis. Our analysis identified 12 metabolites that include flavan (catechin), flavonols (kaempferol, myricetin, quercetin, rutin), phenolic acids (gallic acid, cinnamic acid, caffeic acid, sinapic acid, ferulic acid), flavanones (naringenin) and coumarin. A majority of the polyphenols studied here were found at higher levels in CDC Pintium as compared to 1533–15 (Fig. 3). The concentrations of kaempferol (4 times) and flavan-3-ol catechin (3 times) were significantly higher in CDC Pintium as compared to 1533–15 immature seed coat. Catechin and epicatechin are monomers that are transported to vacuoles where they are polymerized into proanthocyanidins [21]. A 2.9 times higher level of kaempferol has been previously reported in the non-aged mature seed coat of CDC Pintium compared to 1533–15 [9]. The same study showed a 60% reduction in kaempferol level in aged seed coat of CDC Pintium as compared to non-aged. No such change in kaempferol level was seen in 1533–15. Beninger and colleagues [9] also found that a catechin-kaempferol dimer adduct was identified in both of the pintos, where aging increased the amount of adducts by 3 to 5-fold in CDC Pintium. It has been hypothesized that these adducts are formed during the oxidation of proanthocyanidins. Elsadr et al. [3] also previously reported that catechin over-accumulates in developing seed coat of CDC Pintium as compared with 1533–15. As shown in Fig. 3, our results revealed that the production of phenolic acids, starting with *t*-cinnamic acid, the first intermediate after phenylalanine, which feeds into the general phenylpropanoid pathway including the flavonoid pathway, is activated 4 × higher in CDC Pintium compared to 1533–15. Naringenin also accumulated 1.8 × higher in CDC Pintium compared to 1533–15 seed coat. Naringenin synthesis is the first committed step for the production of all flavonoids including proanthocyanidin monomers. Our data demonstrate that the flavonoid pathway, in general, is more active in CDC Pintium than in 1533–15, with naringenin substrate being channelled towards the synthesis of flavonoids, thus increasing the overall metabolic flux.

### Analysis of seed coat transcriptome in SD and RD pinto beans

We demonstrated that developing seed of pinto beans (150 mg seed stage) actively accumulates flavonoids including catechin and its precursors in their seed coat. Therefore, to gain insight into the genes involved in seed coat darkening, we performed RNAseq analysis of the same stage of developing seed coat from SD line 1533–15 and RD line CDC Pintium. In all measures, data appeared abundant, high quality and of a strong representation of the *P. vulgaris* transcriptome (Table 1). Interestingly, nearly all representative transcripts for the transcriptome (91.06%) had reads mapped to them (2432 genes with no reads mapped) in the pooled collection of all biological replicates. Individual replicates had between 75 and 85% of transcript models with reads mapped to them. This seems very high, however, the seed coat is a complex tissue and this may be representative of that complexity. The plot of dispersion estimates for all genes showed a fairly tight distribution as would be expected for good quality data possessing four replicates (Additional file 2: Figure S1).

**Table 1:** RNAseq quality and coverage of bean transcriptome

Cultivars	Replicate	Total reads	Q30 bases (%)	Mapped reads	Uniquely mapped reads	Genes hit (%)
1533–15	1	29,823,880	90.39	20,347,924	11,054,222	78.84
	2	18,850,340	89.45	12,991,256	8,067,224	76.39
	3	53,681,314	87.82	36,390,339	19,694,642	84.75
	4	32,211,588	90.49	21,458,754	9,324,847	74.73
CDC Pintium	1	55,184,220	88.77	35,551,838	16,669,271	78.62
	2	42,822,186	88.32	26,665,236	12,683,699	76.12
	3	22,713,516	88.8	13,754,893	6,909,101	73.25
	4	40,101,598	90.23	24,956,759	10,807,837	74.85

Replicate: the variety replicate sequenced; total reads: the total number of sequence reads obtained; Q30 bases (%): the percentage of bases with > 30 quality in the sequencing results; mapped reads: the number of reads that mapped to one of the *P. vulgaris* transcript models; uniquely mapped reads: number of mapped reads mapping uniquely to a transcript model with a mapping quality ≥20; genes hit: number of gene models with one or more reads mapped to its transcript as a percentage of the total number of gene models

Coverage of pinto bean cv 1533–15 and CDC Pintium seed coat transcriptome ranged from 73.25–84.75%

### Differential gene expression analysis

Before calculating differential expression, an independent filter removing the bottom 10% of transcripts based on total read counts (3186 transcripts including the 2432 genes with no reads mapped mentioned above) was applied in order to simplify the analysis. At a false discovery rate (FDR) of ≤0.001, a total of 922 genes were DE between the two pinto bean lines with 260 genes up-regulated (at least 2× higher) and 203 genes down-regulated (less than 2× lower) in CDC Pintium compared to 1533–15 seed coats (Additional file 3: Table S2). On examination of the Benjamini-Hochberg adjusted *p*-value distribution (Additional file 4: Figure S2A), and the log p-value as a function of all genes ranked by total read counts (Additional file 4: Figure S2B), a large percentage of highly significant genes and a very strong baseline of moderately significant genes were observed. Grouping of log significance scores into 3 levels of significance categorized the DE genes with a strong baseline of 814 significant transcripts (*p* ≤ 0.001), 95 highly significant transcripts (*p* ≤ 1e-15) and a collection of 13 extremely significant transcripts (p ≤ 1e-55) (Additional file 5: Figure S3, Additional file 6: Table S3). As the two lines studied, CDC Pintium and 1533–15, are closely related but not genetically identical, it is possible that the 814 significant transcripts in the lowest level represent expression variation that exists across the baseline functions of seed coat biology between the two varieties that may include the genes that are involved in the slow darkening trait. Further, as the primary phenotypic difference between the two genotypes is the rate at which seed coat darkening occurs, the hypothesis is made that the upper two levels of significant transcripts constitute, at least in part, the components of the metabolic pathways necessary for producing this difference. Regardless, it is important to note that the gene expression observed here in the young seed coat leads to a flavonoid profile that produces the trait postharvest.

**Fig. 4:**
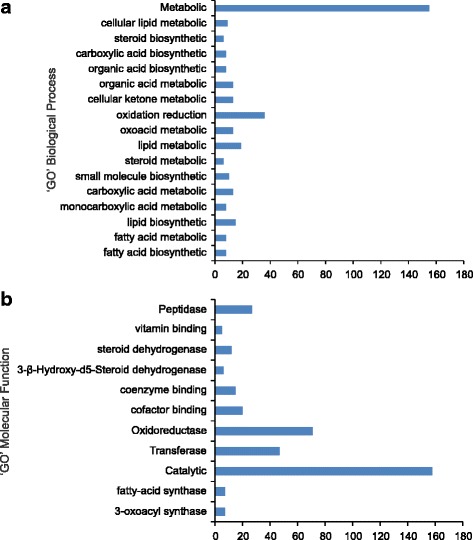
GO enrichment analysis of DE genes in RD and SD pintos. DE genes are categorized into groups according to their (**a**) biological process and (**b**) molecular function

To assess DE genes with respect to their fold change and abundance within each line, a plot of log fold-change against the percentile ranking of total read counts was created with highly significant genes differentially labeled (Additional file 7: Figure S4). Heat maps comparing the relative expression levels of these transcripts across all eight samples (4 biological replicates each) clearly grouped the 8 samples into 2 groups with each group representing either CDC Pintium or 1533–15 (Additional file 8: Figure S5). Additional file 8: Figure S5 also validated similar expression level of genes in each replicate within a line hence increasing the confidence in data quality. Comparison of the differential expression levels (*p* ≤ 1e-15) of the 95 highly significant transcripts between the two varieties revealed that 61 were downregulated and 34 were upregulated in 1533–15 (Additional file 6: Table S3). Gene ontology (GO) enrichment analysis of 922 DE genes revealed that 348 genes were associated with biological processes which included 155 genes involved in metabolic processes and a remaining 193 genes involved in processes concerning fatty acids, small molecules, organic acids, lipids and oxidation reduction (Fig. 4). The GO enrichment analysis also identified 375 genes with molecular function where 158 DE genes may encode for proteins with catalytic activity. The other 213 DE genes under GO molecular function were associated with transferase or oxidoreductase activity, dehydrogenase activity, peptidase activity and vitamin and coenzyme binding (Fig. 4). This analysis also identified several genes that function in flavonoid biosynthesis.

Table 2 lists the 13 most significant genes with very large differences in abundance between the two varieties that encode proteins such as proteases and their inhibitors, endonucleases and hydrolases. Seven of these genes were up-regulated in the 1533–15 samples while the other six were up-regulated in CDC Pintium. Most notable among the list is a membrane protein belonging to the multidrug and toxic compound extrusion (MATE) transporter family and two chalcone synthases (CHSs), the first committed enzyme in the biosynthesis of flavonoids. MATE proteins in prokaryotes and eukaryotes have been known to mediate resistance against diverse drugs and toxic organic effluents [22, 23]. In Arabidopsis, multiple MATE proteins have been characterized with roles including vacuolar transport of proanthocyanidin monomers TT12, [24], control of iron homeostasis FRD3, [25] and lateral root formation ALF5, [26] among others. Since catechin and epicatechin monomers are transported to the site of proanthocyanidin oligomerization in the vacuole with the help of a membrane transporter, the MATE protein encoding transcript identified here may play an important role in the increased accumulation of this compound in pinto bean. Similarly, two CHSs identified here as upregulated in CDC Pintium catalyze the first committed step in flavonoid biosynthesis and divert the metabolic flux of carbon towards the synthesis of a diverse set of metabolites with important roles in flowering plants, such as providing floral pigments, antibiotics, UV protectants and insect repellents, disease resistance and proanthocyanidins [27].

**Table 2:**
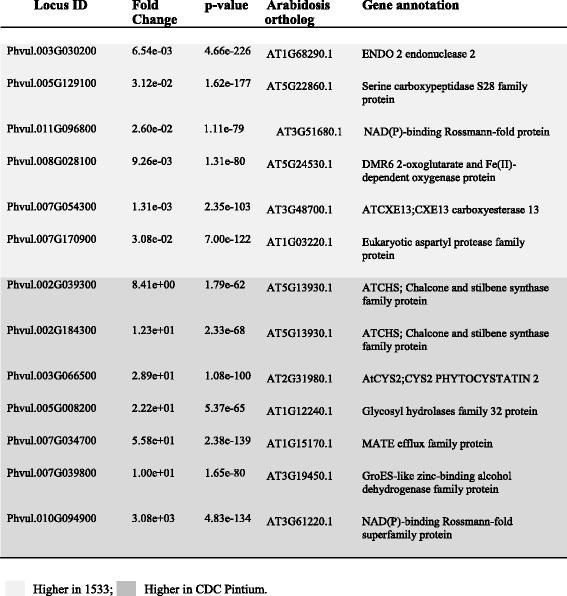
List of extremely significantly (p ≤ 1e-55) differentially expressed genes between CDC Pintium and 1533–15 at stage 150 mg of seed development

**Fig. 5:**
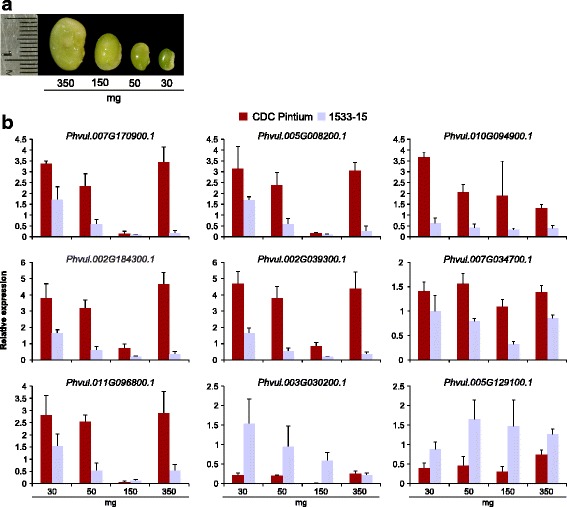
Expression analysis of highly DE genes in RD and SD pinto lines. **a** Four different stages (30, 50, 150 and 350 mg seed weight) of CDC Pintium seeds during development. **b** Total RNA extracted from seed coat was collected from CDC Pintium (RD) and 1533–15 (SD) pinto seeds at the stages shown in A and was used for qRT-PCR analysis using gene-specific primers. Relative expression corresponds to mean value in two biological replicates and 3 technical replicates per biological replicate. Error bar indicates SEM

A qRT-PCR analysis was employed to quantitate the exact transcript accumulation of the most highly significant DE genes in the seed coat tissue collected from both CDC Pintium and 1533–15. The same RNA samples used in constructing the sequencing libraries were utilized for reverse transcription. Interestingly, this not only confirmed the differential gene expression as detected by RNAseq, but also illustrated the expression pattern of those genes during multiple stages of seed coat development (30, 50, 150 and 350 mg; Fig. 5a). In several cases, the largest difference in the expression of the selected genes was observed in seed coat collected from 30 or 350 mg stage of developing seeds (Fig. 5b).

**Fig. 6:**
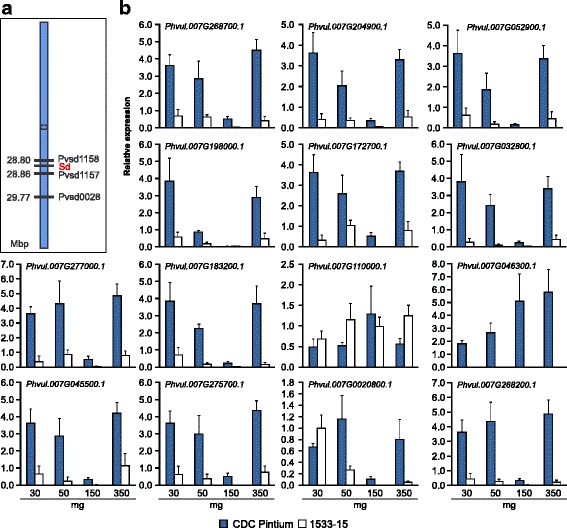
Expression analysis of DE genes located on chromosome 7 in RD and SD pinto lines. **a** A schematic diagram of chromosome 7 in pinto bean showing the location of SSR markers and *Sd* gene. **b** Gene expression analysis of DE genes located on chromosome 7. Total RNA extracted from seed coat was collected from CDC Pintium (RD) and 1533–15 (SD) pinto seeds at 4 different stages of development and was used for qRT-PCR analysis using gene-specific primers. Relative expression corresponds to mean value in two biological replicates and 3 technical replicates per biological replicate. Error bars indicate SEM

Genetic analysis of postharvest darkening of seed coat in pinto beans revealed that the SD trait is controlled by a single gene, *Sd,* with recessive inheritance [4]. An allelism test conducted with three SD pinto bean lines 1533–15, Saltillo [28] and SDIP-1 [29] suggested that they carry the same *sd* gene [3]. A single nucleotide polymorphism assay in a RIL mapping population generated from a cross between CDC Pintium and 1533–15 demonstrated that *Sd* is linked with two simple sequence repeat (SSR) markers: Pvsd-1157 and Pvsd-1158 Fig. 6a; [11], located on chromosome 7. An attempt to search for the genes located in between the two SSR markers identified only 2 genes: *Phvul.007G171800* and *Phvul.007G171900,* with the functional annotations of ras homolog gene family member T1 and fantastic four meristem regulator, respectively. No difference in the expression of these two genes was detected between CDC Pintium and 1533–15. However, our RNAseq analysis identified 17 DE genes on chromosome 7 where 11 genes were down- and 6 genes were up-regulated in 1533–15 relative to CDC Pintium. Among these, genes encoding an amino acid transporter (*Phvul.007G032800*), histidine containing phosphotransmitter (*Phvul.007G183200*), and a hydrolase superfamily gene (*Phvul.007G198000*) were up-regulated while genes such as the previously mentioned MATE efflux family protein (*Phvul.007G034700*) and protein kinase (*Phvul.007G268200*) were down-regulated in 1533–15 compared to CDC Pintium. Analysis of DE genes located on chromosome 7 during seed coat development by RT-qPCR revealed some interesting features (Fig. 6b). First of all, even though DE genes were detected by RNAseq in the seed coat from 150 mg stage pinto bean seed, as in the previous analysis, higher transcript abundance and larger differences in transcript level between CDC Pintium and 1533–15 were observed in younger or older seed coats; this is consistent with the fact that metabolic activity is lowest at the 150 mg stage, which is dominated by storage product accumulation, as determined by in silico analysis of seed ESTs during development [30]. Additionally, our qRT-PCR analysis did not correlate with RNAseq analysis for some of the genes genes. RNAseq analysis suggested that genes *Phvul.007G198000* (*p* ≤ 1e-15), *Phvul.007G032800* (*p* ≤ 1e-15) and *Phvul.007G183200* (*p* ≤ 1e-15) were expressed at higher levels in 1533–15 compared to CDC Pintium, but no such difference was observed through qRT-PCR analysis in the same set of tissue samples. One common feature of these genes is they belong to large gene families. Even though RNAseq is a powerful technology, its limitations due to gene family sizes, overlapping gene models, different gene isoforms and genes with short transcripts can make the interpretation of data difficult [31]. Therefore, the DE genes identified require detailed characterization to investigate their direct or indirect role in postharvest seed coat darkening.

### Expression analysis of phenylpropanoid genes

**Fig. 7:**
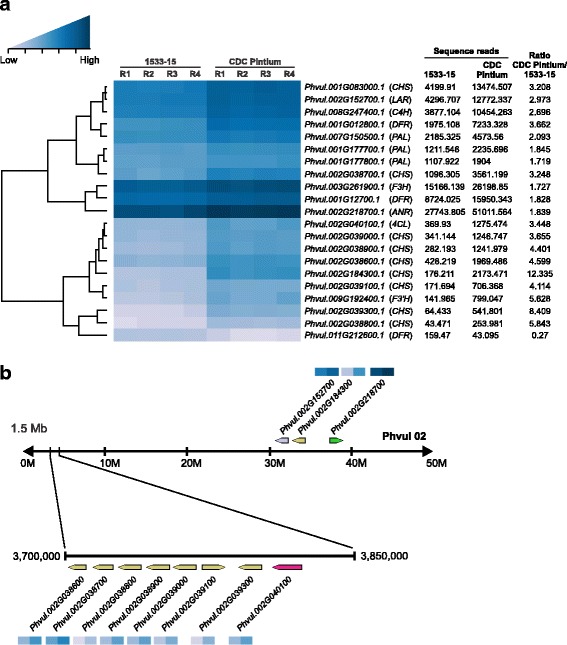
Heatmap and genomic organization of DE phenylpropanoid genes. **a** Four replicates (R1-R4) of 150 mg stage seed coats from CDC Pintium and 1533–15 showing expression levels of DE phenylpropanoid genes. The numbers indicate transcript counts. Locus IDs are shown in the right and their corresponding annotations are in the parenthesis. The color scale above the heat map indicates expression levels low transcript abundance (light blue) to high transcript abundance (dark blue). **b** DE phenylpropanoid genes were clustered in a 1.5 Mb region of the proximal region and an 8 Mb distal end of chromosome 2. Enlargement of 1.5 Mb region shows multiple *CHS* gene family members. Arrows pointing left or right indicate reverse or forward orientation of the gene in the chromosome. Members of the same gene family are shown in same color. Heatmap indicates mean expression levels (color scale as in (**a**)) of the genes in 1533–15 (left) and CDC Pintium (right)

To study the expression profile of phenylpropanoid genes in pinto beans, we searched the *P. vulgaris* genome (https://phytozome.jgi.doe.gov/pz/portal.html#!info?alias=Org_Pvulgaris) for genes involved in flavonoid biosynthesis. This search identified a total of 59 loci encoding 12 genes (Additional file 9: Table S4). Most of the upstream phenylpropanoid pathway genes are represented by large gene families such as *phenylalanine ammonia lyase* (*PAL*), *cinnamate-4-hydroxylase* (*C4H*), *4-coumarate:CoA ligase* (*4CL*) and *CHS*. The genes functioning downstream of the pathway are from small gene families or are just a unique gene. Figure 7a displays a heat map of 21 phenylpropanoid genes DE between the RD and SD pintos based on the RNAseq data. Except for *Phvul.011G212600,* which encodes for dihydroflavonol 4-reductase (DFR), all other genes were expressed at lower level in 1533–15 compared to CDC Pintium. The difference in expression between the pintos was consistent among the replicates within a line (Fig. 7a). A search for *CHS* genes in *P. vulgaris* genome identified at least 14 putative genes. Our transcriptome analysis revealed that 9 *CHS* genes (*Phvul.001G083000, Phvul.002G038600, Phvul.002G038700, Phvul.002G038800, Phvul.002G038900, Phvul.002G039000, Phvul.002G039100, Phvul.002G039300* and *Phvul.002G184300*) were highly expressed (3.2 to 12.3 times higher) in CDC Pintium compared to 1533–15 (Fig. 7a). Except for *Phvul.001G083000,* the other DE *CHS* are located in a tandem arrangement on chromosome 2 where 7 *CHS* gene family members and a *4CL* are clustered in a 1.5 Mb proximal region (Fig. 7b). An 8 Mb distal region of chromosome 2 contains a DE *CHS* (*Phvul.002G184300*, 12.3×) and two downstream proanthocyanidin genes, *LAR* (*Phvul.002G152700*) and *ANR* (*Phvul.002G218700*). LAR is the first committed enzyme in the biosynthesis of proanthocyanidin monomer catechin which diverts leucocyanidin towards catechin biosynthesis. ANR catalyzes a similar reaction with cyanidin forming epicatechin. *LAR* transcript level was 2.97 times higher in CDC Pintium compared to 1533–15. LAR competes with ANS for the substrate leucocyanidin which will then either lead to proanthocyanidin or anthocyanin biosynthesis (Fig. 2). Other proanthocyanidin genes such as *F3H* (*Phvul.003G261900*) and *DFR* (*Phvul.001G012700*) were also expressed at lower levels in 1533–15 compared to CDC Pintium (Fig. 7a). Our transcriptome analysis reveals the upregulation of the entire proanthocyanidin biosynthetic pathway from *PAL* to *LAR*/*ANR* except for *CHI* in CDC Pintium vs 1533–15, suggesting that downregulation of proanthocyanidin genes in 1533–15 seed coat pre-conditions the SD trait. These data combined with the genetic analysis [4] suggests that there may be a regulator gene on chromosome 7 which may be co-regulating multiple genes in proanthocyanidin pathway.

## Conclusion

**Fig. 8:**
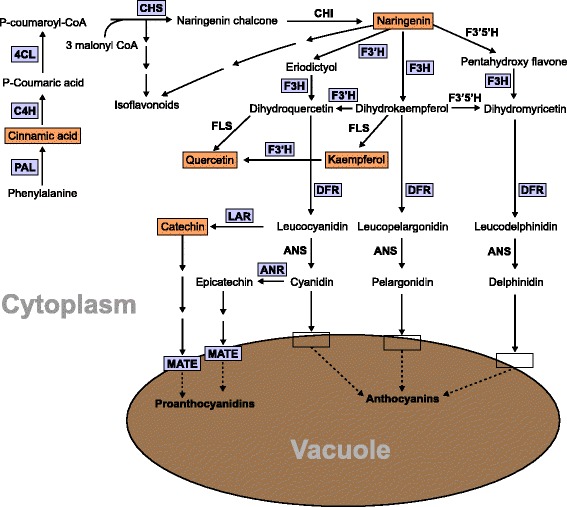
Summary of proanthocyanidin biosynthetic gene transcript and metabolite accumulations in SD and RD pinto bean seed coats. Transcripts down regulated in SD pinto bean 1533–15 compared to RD CDC Pintium are highlighted in purple. Reduced accumulations of metabolites in 1533–15 compared to CDC Pintium are shown in orange highlight. Full name of pathway enzymes are in Fig. 2

Our analysis of flavonoids in pinto bean immature seed coat showed that kaempferol and catechin were produced at significantly higher levels in CDC Pintium (RD) compared to 1533–15 (SD) line. The transcriptome analysis of developing seed coats in these pintos identified several highly DE phenylpropanoid and transporter genes. As shown in Fig. 8, our combined analysis of metabolic and transcriptome data suggests a down-regulation of metabolic flux for reduced production of proanthocyanidin monomers and their transport in 1533–15 compared to CDC Pintium. However, these phenylpropanoid genes are not located on chromosome 7 and, therefore, are not the candidate *Sd* gene, except for the MATE transporter, which is outside of the interval defined by the SSR markers. It is possible that differential expression of structural genes or regulators was not detected due to developmental stage specific expression. Analysis of gene expression of the candidate genes during multiple stages of seed coat development may help identify the *Sd* gene if it is involved in flavonoid regulation. However, it is more likely that the regulator was not identified in RNAseq due to the lack of differential expression. Additionally, the potential role of the MATE transporter can be investigated to study how it affects the transport and accumulation of proanthocyanidin precursors into the vacuoles, thus affecting overall proanthocyanidin accumulation in RD and SD pinto bean lines.

The dataset generated in this study provides a significant resource for further molecular and biochemical studies of postharvest seed coat darkening in pinto beans.

## Additional files

Additional file 1: **Table S1.** Primers used in quantitative RT-PCR verification of DE genes. (DOCX 20 kb)

Additional file 2: **Figure S1.** Estimated dispersion of all genes. Dispersion values (y-axis) plotted as a function of expression strength for each gene as returned by DESeq. (PDF 481 kb)

Additional file 3: **Table S2.** List of 922 DE genes (*p* < 0.001) in CDC Pintium and 1533–15. (XLSX 137 kb)

Additional file 4: **Figure S2.** Histogram of *p*-values and MA plot of log_2_ fold change vs average expression of each gene. Significantly DE genes (FDR ≤ 0.001) were assessed for significant differential expression between the two cultivars of 1533 and CDC. (PDF 714 kb)

Additional file 5: **Figure S3.** Plots of ranked average gene counts against –log_10_ of the *P*-value. Significant scores are divided into three levels showing a baseline, high and extremely significant levels of differential expression. (PDF 627 kb)

Additional file 6: **Table S3.** List of 85 DE genes, 61 upregulated and 34 down regulated in CDC Pintium vs 1533–15. (XLSX 28 kb)

Additional file 7: **Figure S4.** Plot of ranked average gene counts against log_10_ fold change. Genes with high significance (*p* ≤ 1e-15) are labeled in blue, genes with extreme significance (p ≤ 1e-55) are labeled in red with their corresponding gene identifier provided. (PDF 950 kb)

Additional file 8: **Figure S5.** Heatmap of DE genes. Expression of genes A) upregulated and B) down regulated in CDC Pintium vs 1533–15 in each biological replicate. (PDF 566 kb)

Additional file 9: **Table S4.** List of phenylpropanoid genes that were used to search for differential expression. (XLSX 15 kb)

## Abbreviations

CHS: Chalcone synthase
DE: Differential expression
GO: Gene ontology
RD: Regular darkening
SD: Slow darkening
