# Supplementary material for: Slow darkening of pinto bean seed coat is associated with significant metabolite and transcript differences related to proanthocyanidin biosynthesis
Source: BMC Genomics. 2018 Apr 16;19:260. doi: 10.1186/s12864-018-4550-z (PMC5903001; doi:10.1186/s12864-018-4550-z)
Supplement: Supplementary file 1 — Table S1. Primers used in quantitative RT-PCR verification of DE genes. (DOCX 20 kb) [file 12864_2018_4550_MOESM1_ESM.docx]

| **Genes** | **Primer names** | **Sequences (5'-3')** | **Amplicon sizes** |  |
| --- | --- | --- | --- | --- |
| *UBI* | qUBi-F | ACAGCTGGAGGATGAAAGGA | 150 | Reference gene |
|  | qUbi-R, | GTCCGAACTCTCCACCTCAA |  |  |
| *Phvul.004G152500.1* | MATE4qF | TGT GTT CTG TGT GTT GAG TAG C | 261 | Highly differentially expressed genes |
|  | MATE4qR | AGG CCC TGA AAG CCA TAA CTG |  |  |
| *Phvul.006G028700.1* | MATE6qF | TGG AGG TAA CAA GCA TGA ACT C | 170 |  |
|  | MATE6qR | TTG CTT GGG AGT CAA GGC TC |  |  |
| *Phvul.007G034700.1* | MATE7qF | AAACATGGAGGAAAGTCTACTA | 224 |  |
|  | MATE7qR | CAGCTAGGGAAATGGCTAAG |  |  |
| *Phvul.008G197000.1* | MATE8qF | CCTCACTCCATGCACTCCAAC | 225 |  |
|  | MATE8qR | GCC TTG ACT CCC ACA GAA CAA |  |  |
| *Phvul.002G184300.1* | CHS2a-F | AGCACATGACCGAGCTCAAA | 237 |  |
|  | CHS2a-R | GCGTAATCTTTGACTTTGGC |  |  |
| *Phvul.002G039300.1* | CHS2b-F | TGGTGAGTGTATCCGAGATT | 222 |  |
|  | CHS2b-R | GTCTTGCCTCGCATCTAAAG |  |  |
| *Phvul.010G094900.1* | NAD11F | GTTCAACAACGCCGGCATAA | 249 |  |
|  | NAD11R | TTTCTCGTCAGTCCCACCAC |  |  |
| *Phvul.005G008200.1* | GLY5F | ATTGCAACAATCACAAACCCCA | 200 |  |
|  | GLY5R | GATCAATGCAAACAATGACATGAGA |  |  |
| *Phvul.003G030200.1* | ASP7F | CAGCTGACTTTTATTGCAAGCAC | 210 |  |
|  | ASP7R | GCAGTAAGCCAAAGACAACTCA |  |  |
| *Phvul.003G030200.1* | ENDO2-3F | TCGCCTGAGCAGTGCAG | 171 |  |
|  | ENDO2-3R | GTGTGATCGTAACTGCAAACGG |  |  |
| *Phvul.001G103300.1* | MATE1qF1 | AGG TGG TCG AAG GAG AGT GC | 272 |  |
|  | MATE1qR1 | CGA AGC AAA TTG GCA TTC TTA CGA GT |  |  |
| *Phvul.007G172700.1* | qPv07-F | GTTCATGTTTCAAACATGCATTTCAAG | 85 | Differentially expressed genes located on chromosome 7 |
|  | qPv07-R | GAGAAGAAGCCCATGTTAGTGTG |  |  |
| *Phvul.007G032800.1* | qPv07-1-F | GCTTCACCAAATCATAATGGTTTCTG | 109 |  |
|  | qPv07-1-R | GTAACCGAGTTCTTTGAGACGG |  |  |
| *Phvul.007G110000.1* | qPv07-2-F | TGTCCGAGTTTCTCACAGGATTG | 122 |  |
|  | qPv07-2-R | CAGATATGACAGCAACCGATAAAGG |  |  |
| *Phvul.007G183200.1* | qPvu07-3-F | GCAACAAGTGACACAAGAGTACTACTTG | 119 |  |
|  | qPvu07-3-R | TCTGCCTCCTATCACACTACCT |  |  |
| *Phvul.007G198000.1* | qPv07-4-F | CCAACTCTGGAGGAAGTGGTG | 128 |  |
|  | qPv07-4-R | TCTGACGCAGAAACCGTGAC |  |  |
| *Phvul.007G020800.1* | qPv07-5-F | GAGAGTGAAAGTGCTGAGTGATGA | 159 |  |
|  | qPv07-5-R | CCACAGGGCTTATGAAGGGTATC |  |  |
| *Phvul.007G045500.1* | qPv07-6-F | TGATTCAGGAGTGGGGAACTGA | 82 |  |
|  | qPv07-6-R | TCTCCATGGTGCAGTGAAATATCC |  |  |
| *Phvul.007G046300.1* | qPv07-7-F | GGTCCTGGTTGTTTAGAAGGATGC | 135 |  |
|  | qPv07-7-R | GCGATATTCCTCATGTTCAAAGCCA |  |  |
| *Phvul.007G052900.1* | qPv07-8-F | CATCACATACCTTTTCTGTTTCACC | 117 |  |
|  | qPv07-8-R | CACACAAACACAGACATAACAGGT |  |  |
| *Phvul.007G204900.1* | qPv07-9-F | TCAGTGACTCTCATGCTCTCC | 145 |  |
|  | qPv07-9-R | TCTTGAAGTGTGTTGTAAACCTGG |  |  |
| *Phvul.007G268200.1* | qPv07-10-F | CGCTTGTTGAGAAATGGAAACCAC | 159 |  |
|  | qPv07-10-R | CAGGGATACTACTCTTACTGTCCTG |  |  |
| *Phvul.007G268700.1* | qPv07-11-F | TTCAACAGTTCTAGCACCAAGG | 132 |  |
|  | qPv07-11-R | CTCTGTGGACACTCAGAAATGG |  |  |
| *Phvul.007G275700.1* | qPv07-12-F | TGCCTATCCTTGATGGTTGGA | 107 |  |
|  | qPv07-12-R | CGAGACCATGTTTTGTGATGAGAC |  |  |
| *Phvul.007G277000.1* | qPv07-13-F | GGCATTTCGTTCATCAAGAGAC | 101 |  |
|  | qPv07-13-R | CGTCAAAATCTTATCATCTGTTGATCCT |  |  |

**Table S1.** Primers used in quantitative RT-PCR verification of differentially expressed genes.
