# Supplementary figures and images for: Slow darkening of pinto bean seed coat is associated with significant metabolite and transcript differences related to proanthocyanidin biosynthesis
Source: BMC Genomics. 2018 Apr 16;19:260. doi: 10.1186/s12864-018-4550-z (PMC5903001; doi:10.1186/s12864-018-4550-z)

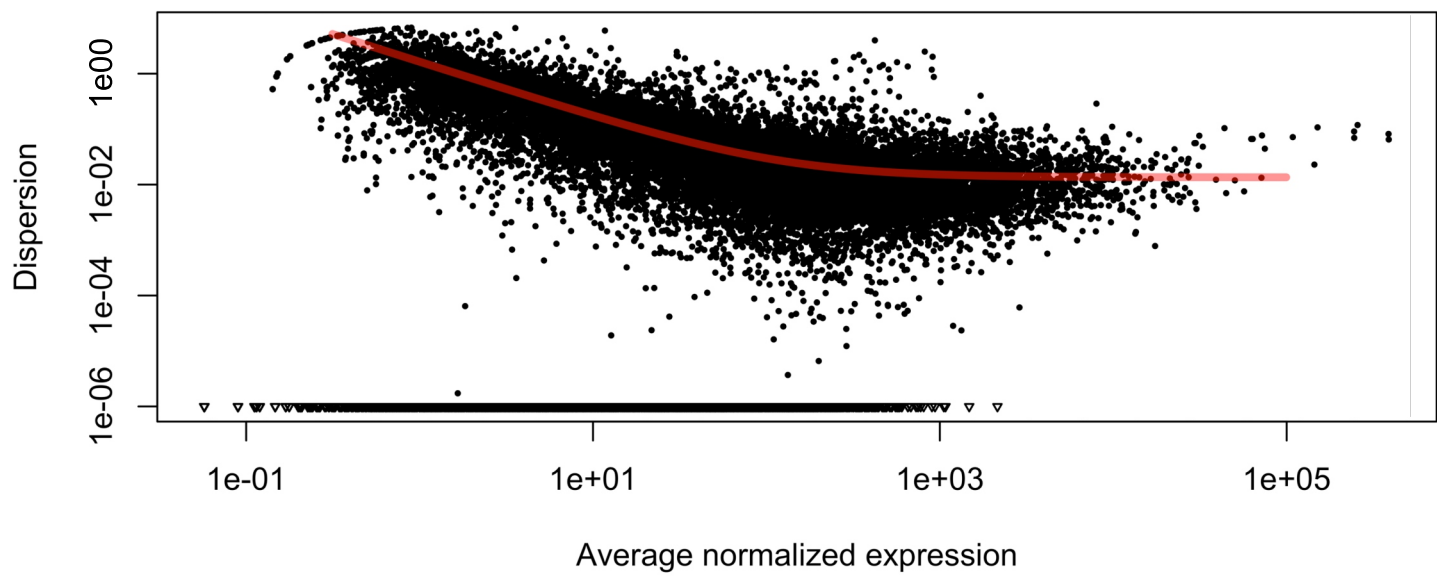

Supplement: Supplementary file 2 — Figure S1. Estimated dispersion of all genes. Dispersion values (y-axis) plotted as a function of expression strength for each gene as returned by DESeq. (PDF 481 kb) [file 12864_2018_4550_MOESM2_ESM.pdf]

**A**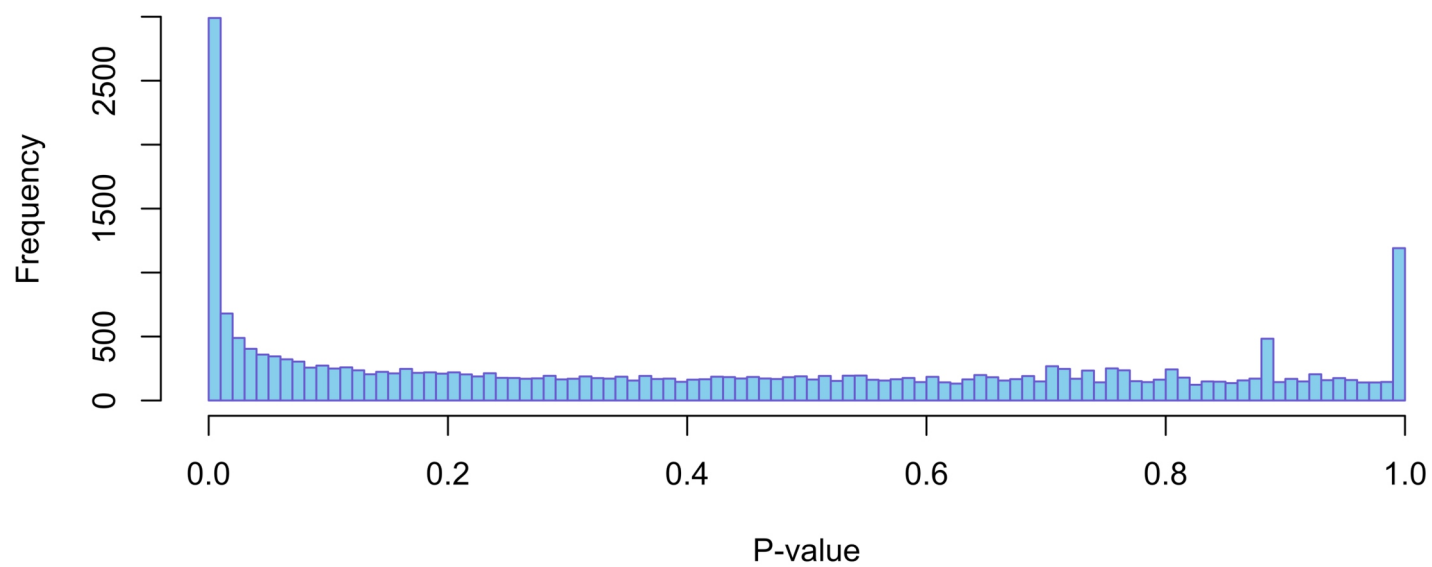**B**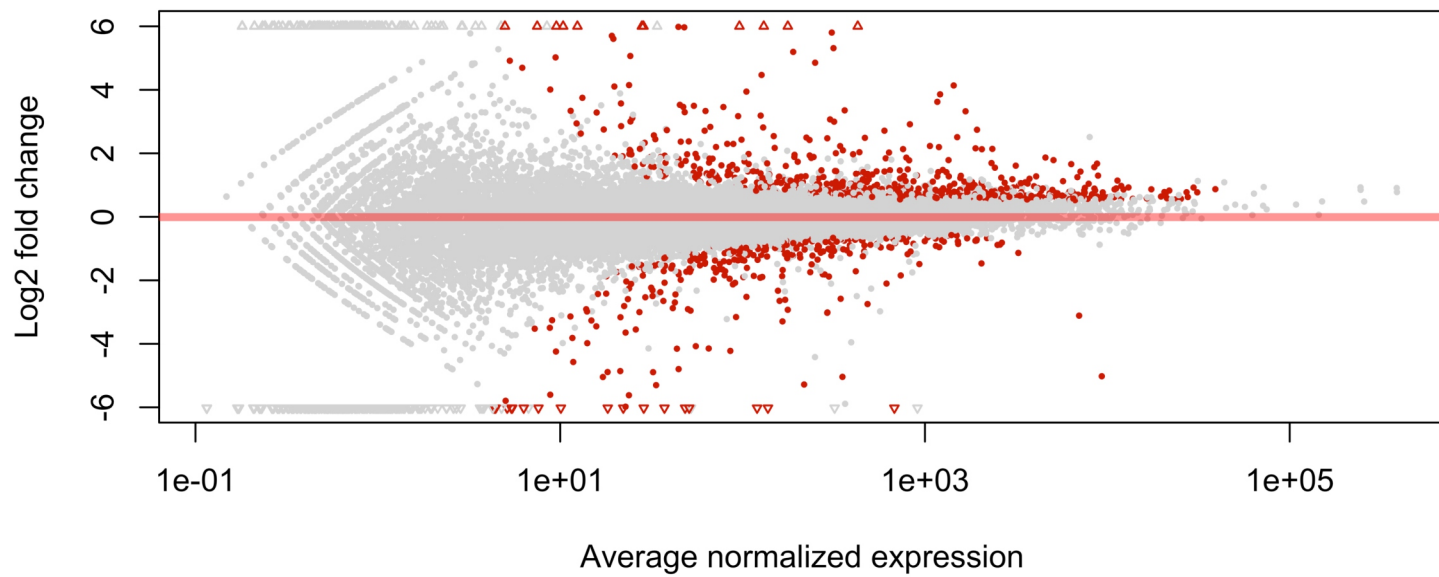

Supplement: Supplementary file 4 — Figure S2. Histogram of p-values and MA plot of log2 fold change vs average expression of each gene. Significantly DE genes (FDR ≤ 0.001) were assessed for significant differential expression between the two cultivars of 1533 and CDC. (PDF 714 kb) [file 12864_2018_4550_MOESM4_ESM.pdf]

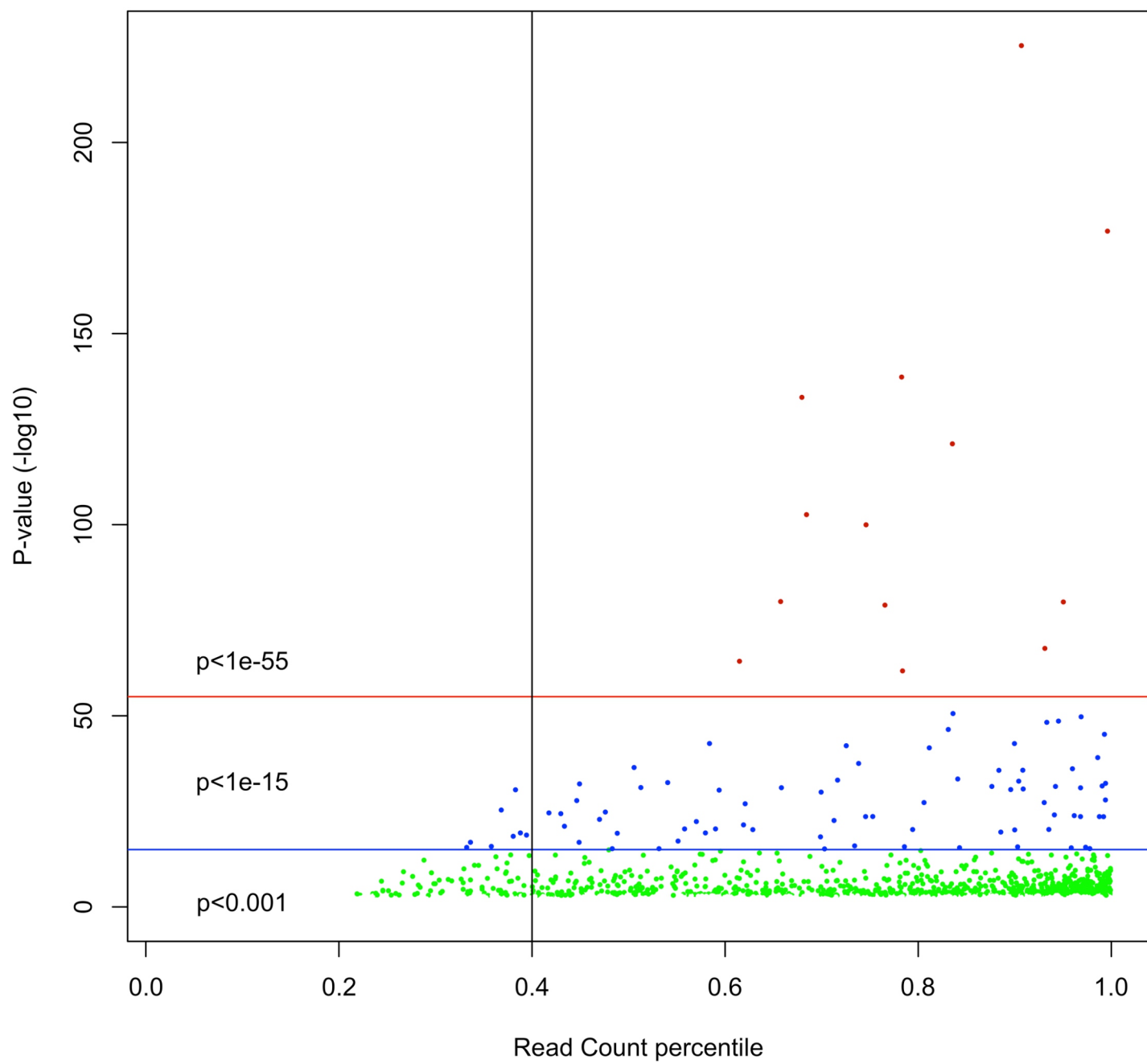

Supplement: Supplementary file 5 — Figure S3. Plots of ranked average gene counts against –log10 of the P-value. Significant scores are divided into three levels showing a baseline, high and extremely significant levels of differential expression. (PDF 627 kb) [file 12864_2018_4550_MOESM5_ESM.pdf]

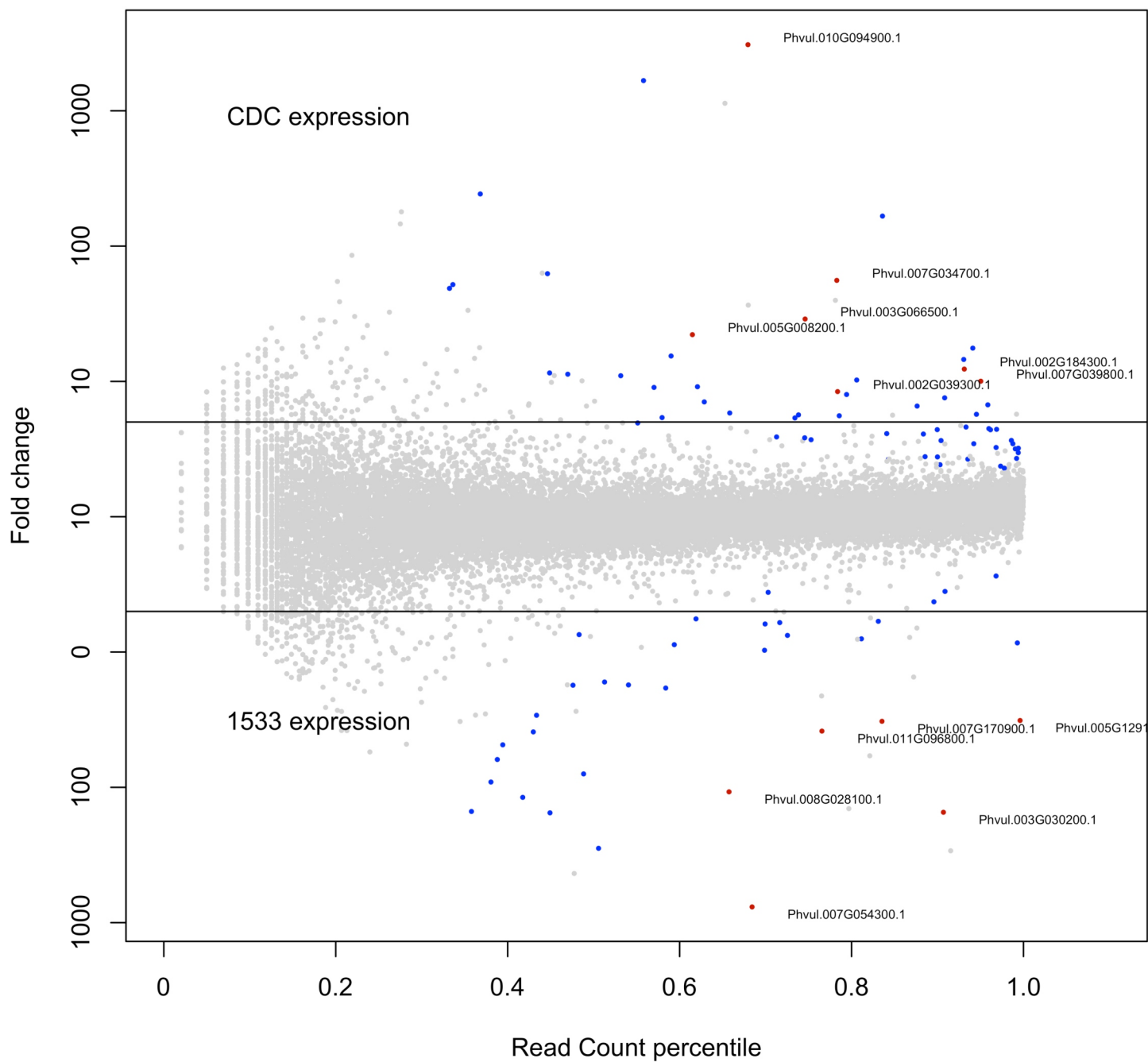

Supplement: Supplementary file 7 — Figure S4. Plot of ranked average gene counts against log10 fold change. Genes with high significance (p ≤ 1e-15) are labeled in blue, genes with extreme significance (p ≤ 1e-55) are labeled in red with their corresponding gene identifier provided. (PDF 950 kb) [file 12864_2018_4550_MOESM7_ESM.pdf]

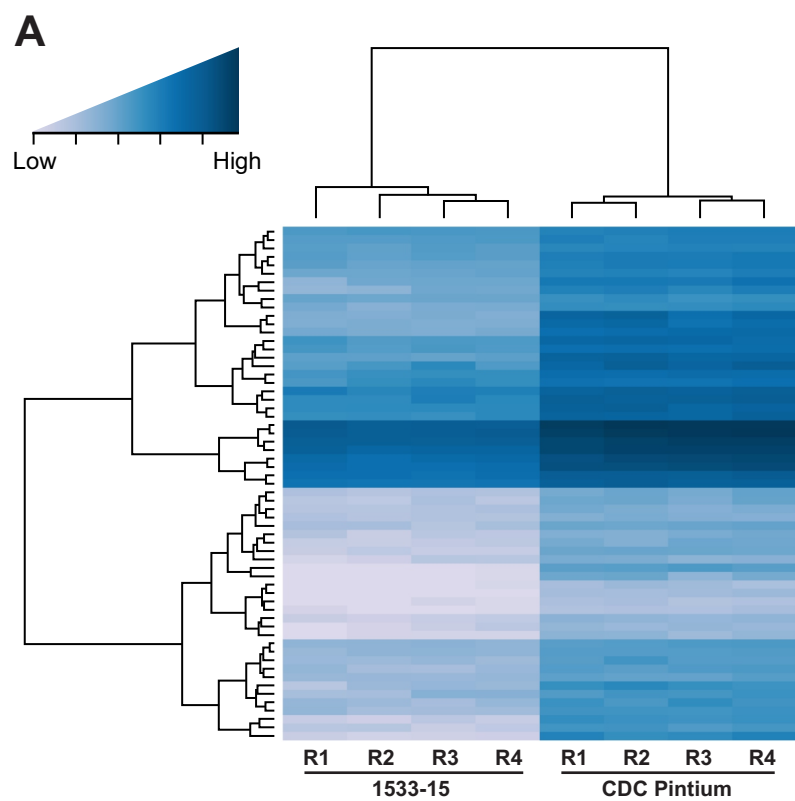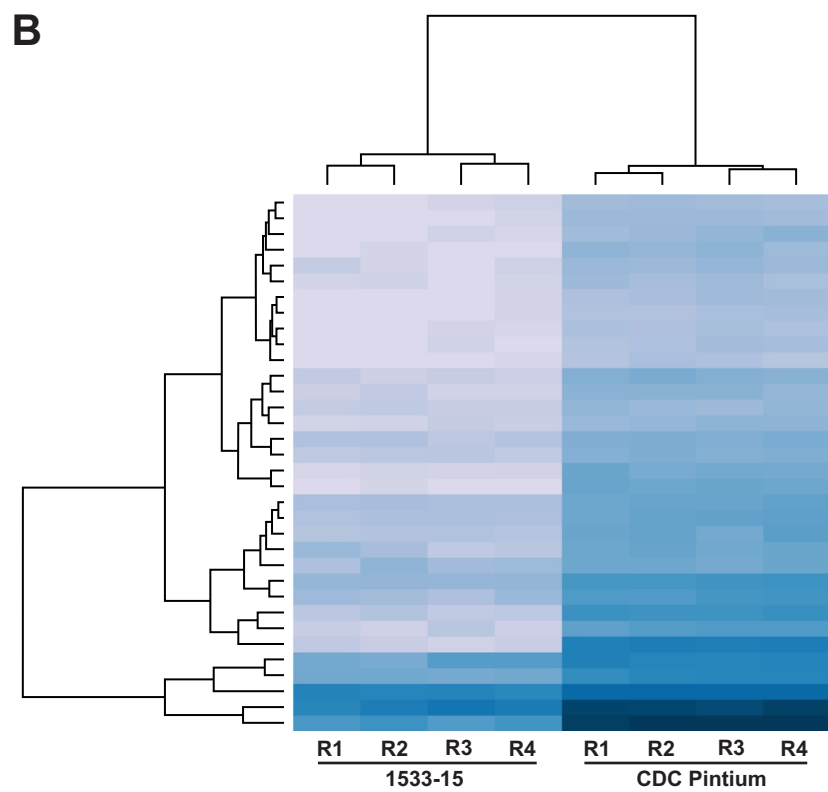

**Figure S5**

Supplement: Supplementary file 8 — Figure S5. Heatmap of DE genes. Expression of genes A) upregulated and B) down regulated in CDC Pintium vs 1533–15 in each biological replicate. (PDF 566 kb) [file 12864_2018_4550_MOESM8_ESM.pdf]
